# Supplementary material for: Effect of Sex and Cross-Sex Hormone Treatment on Renal Monocarboxylate-Transporter Expression in Rats
Source: Pharmaceutics. 2023 Sep 29;15(10):2404. doi: 10.3390/pharmaceutics15102404 (PMC10610497; doi:10.3390/pharmaceutics15102404)

MCT1

OVX Estrogen, OVX Estrogen Placebo

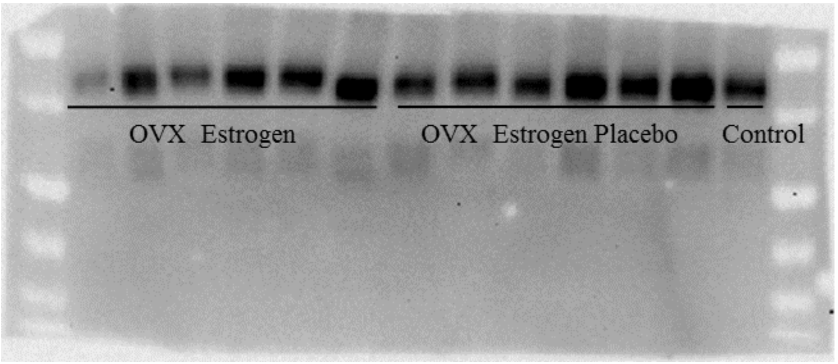

CST Estrogen, CST Estrogen Placebo

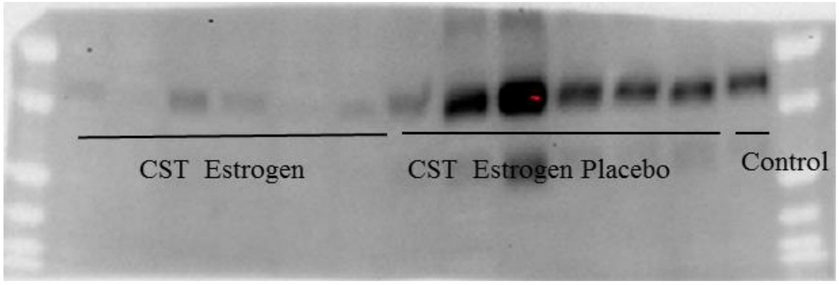

OVX Progesterone, OVX Progesterone Placebo

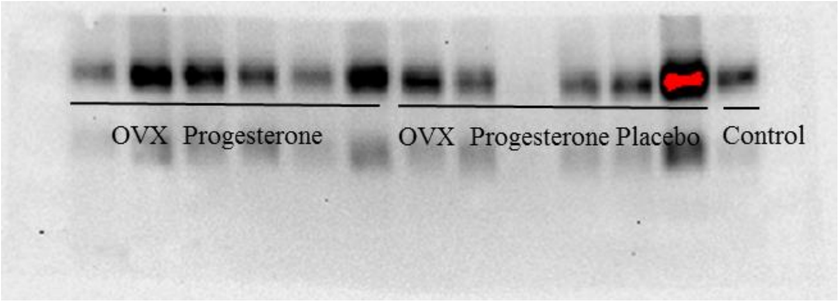

CST Progesterone, CST Progesterone Placebo

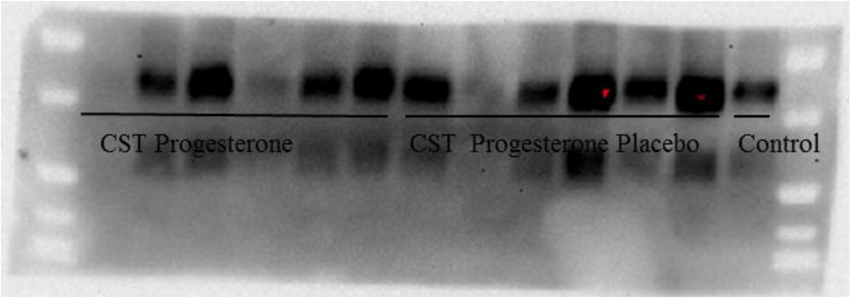

OVX Combo, OVX Combo Placebo

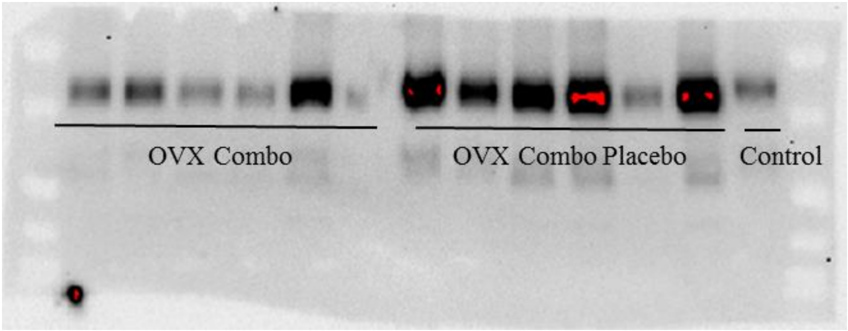

CST Combo, CST Combo Placebo

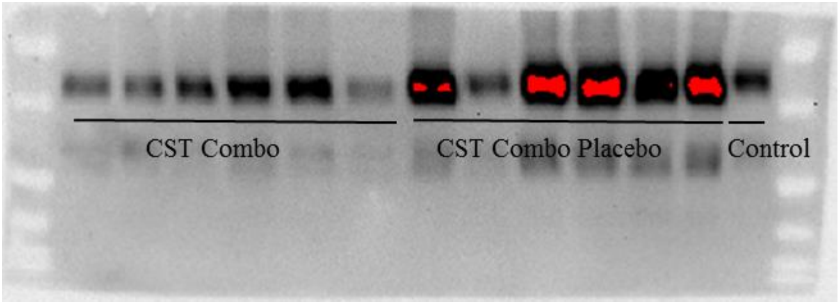

OVX Testosterone, OVX Testosterone Placebo

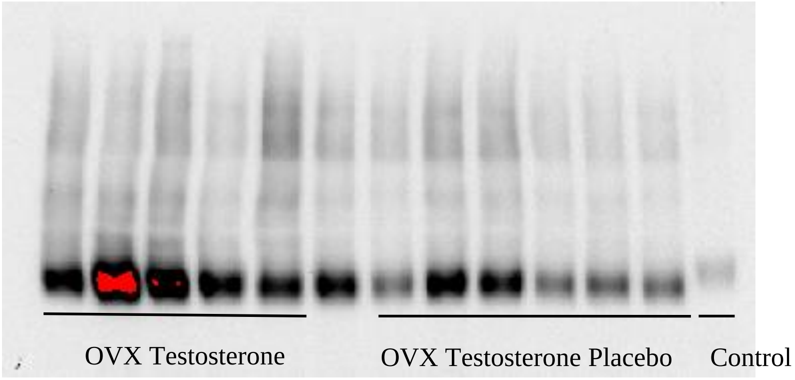

CST Testosterone, CST Testosterone Placebo

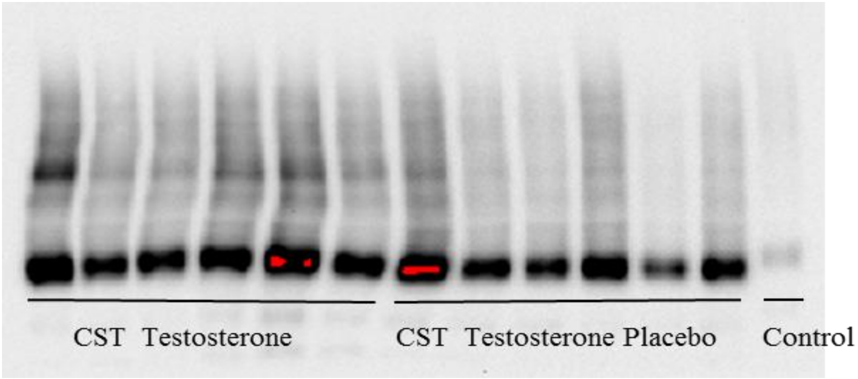

Male, Female

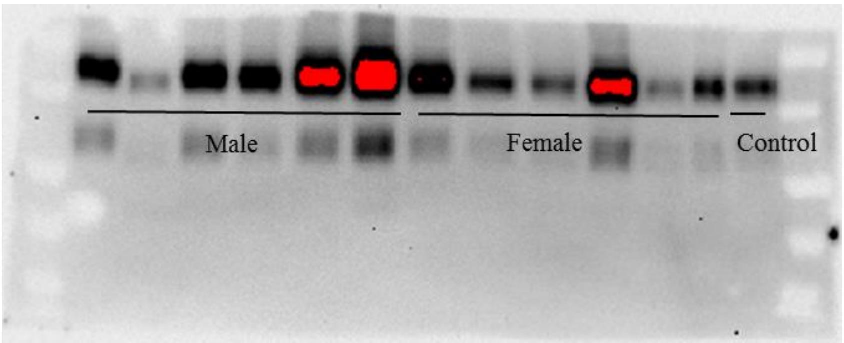

MCT4

OVX Estrogen, OVX Estrogen Placebo

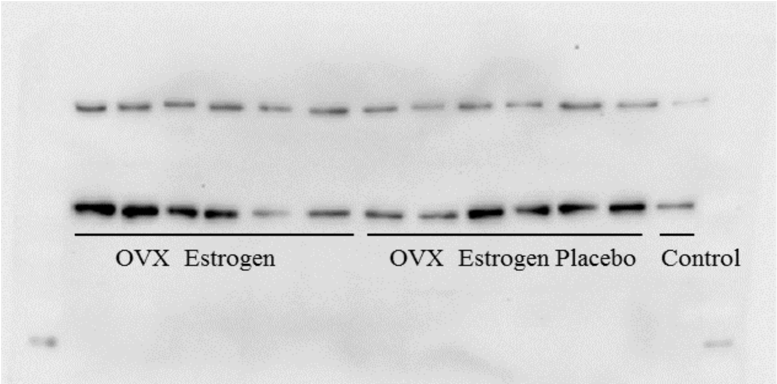

CST Estrogen, CST Estrogen Placebo

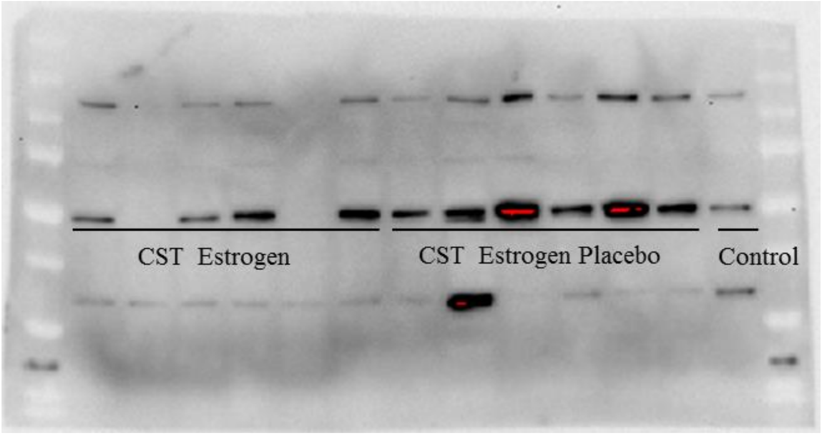

OVX Progesterone, OVX Progesterone Placebo

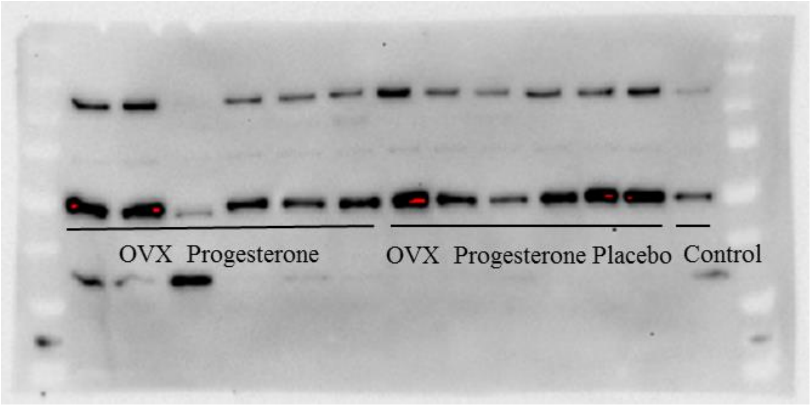

CST Progesterone, CST Progesterone Placebo

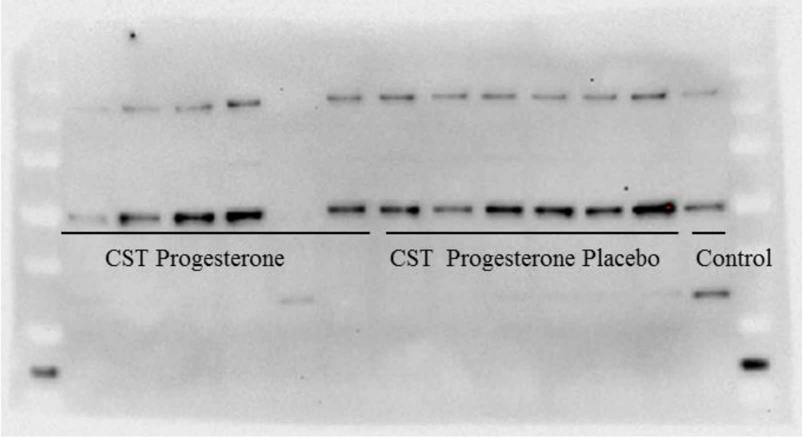

OVX Combo, OVX Combo Placebo

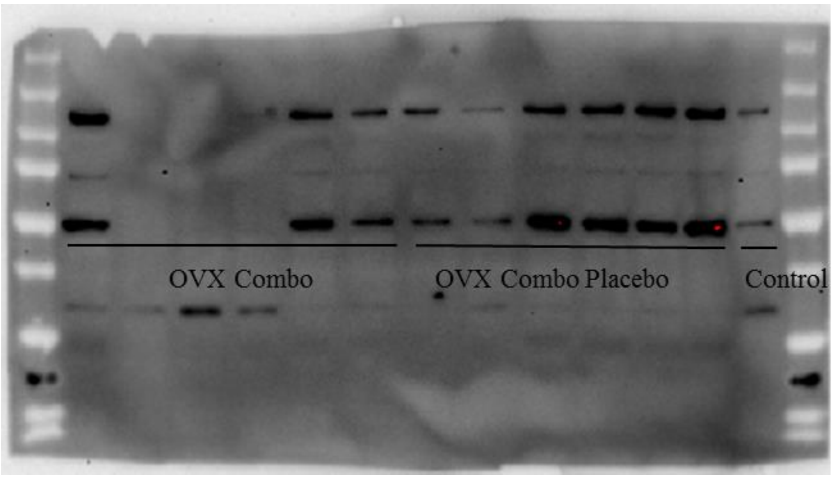

CST Combo, CST Combo Placebo

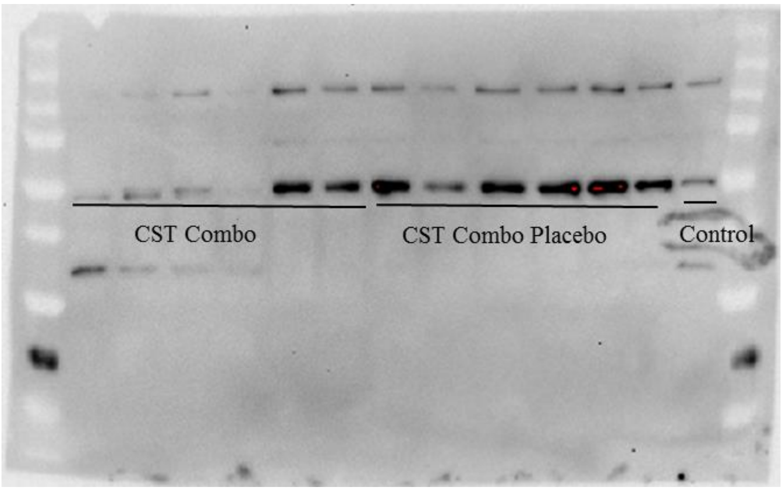

OVX Testosterone, OVX Testosterone Placebo

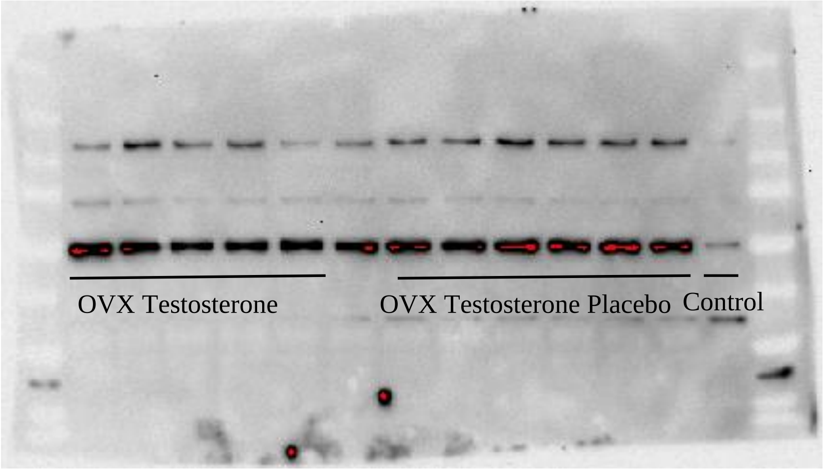

CST Testosterone, CST Testosterone Placebo

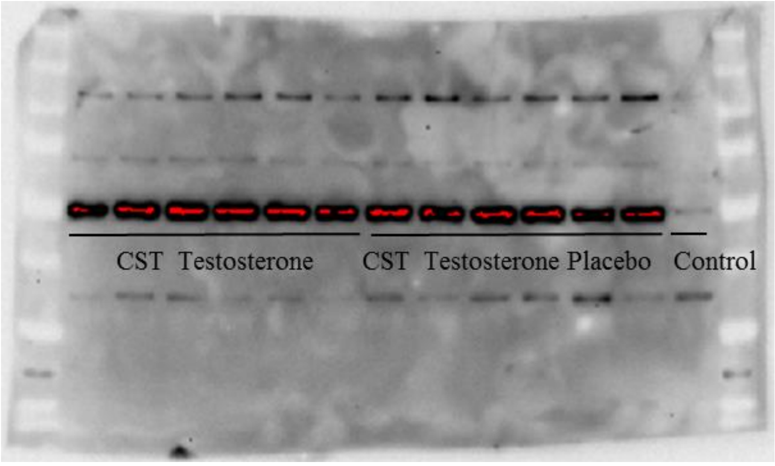

Male, Female

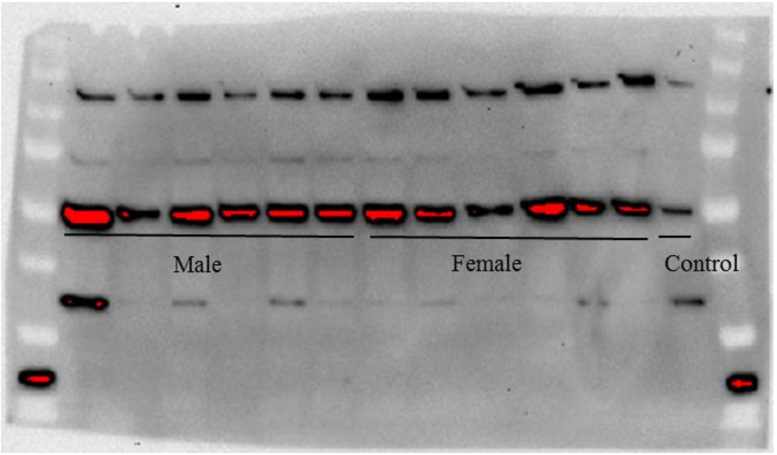

CD147

OVX Estrogen, OVX Estrogen Placebo

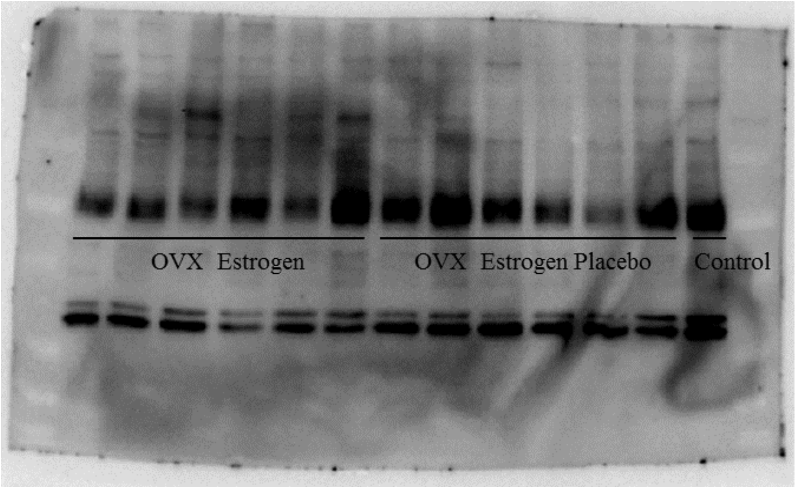

CST Estrogen, CST Estrogen Placebo

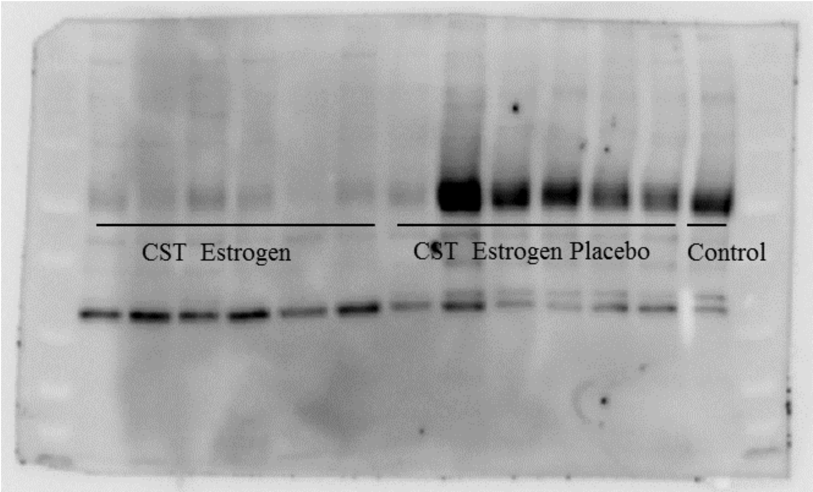

OVX Progesterone, OVX Progesterone Placebo

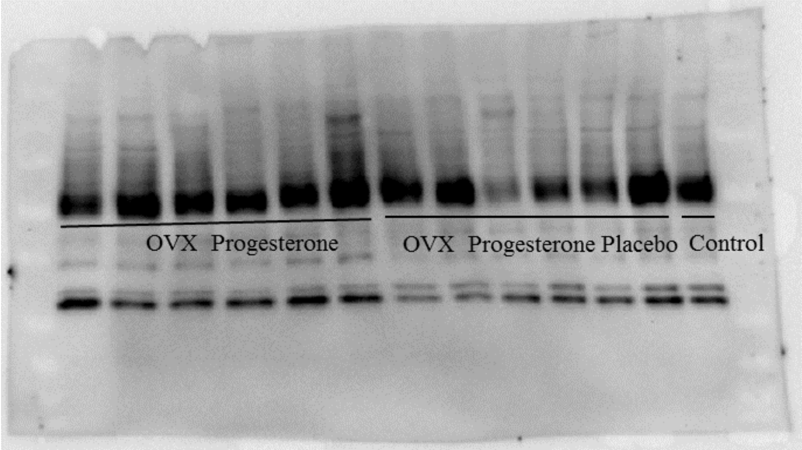

CST Progesterone, CST Progesterone Placebo

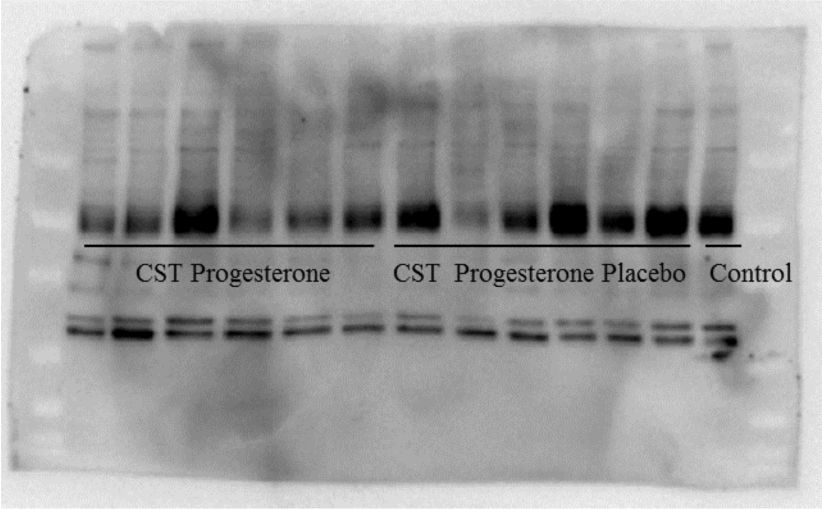

OVX Combo, OVX Combo Placebo

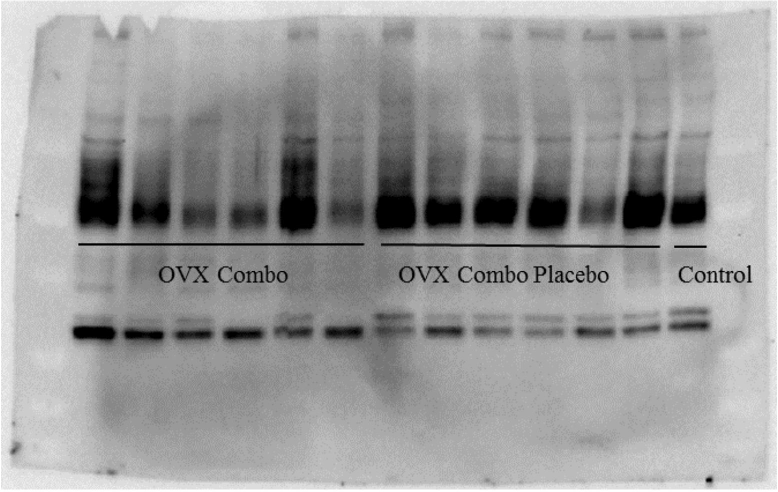

CST Combo, CST Combo Placebo

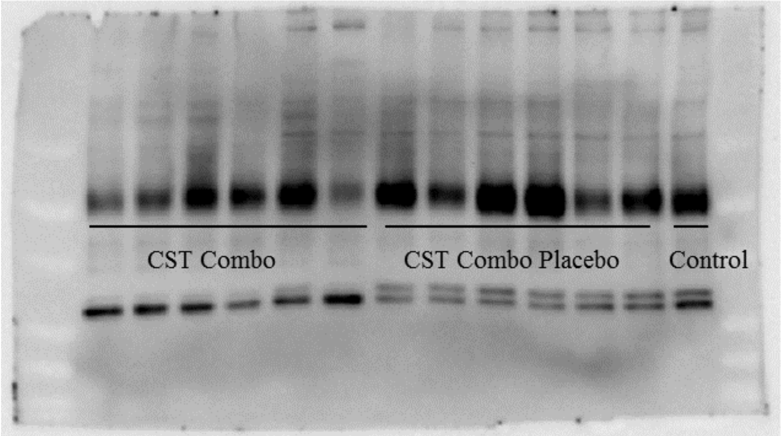

OVX Testosterone, OVX Testosterone Placebo

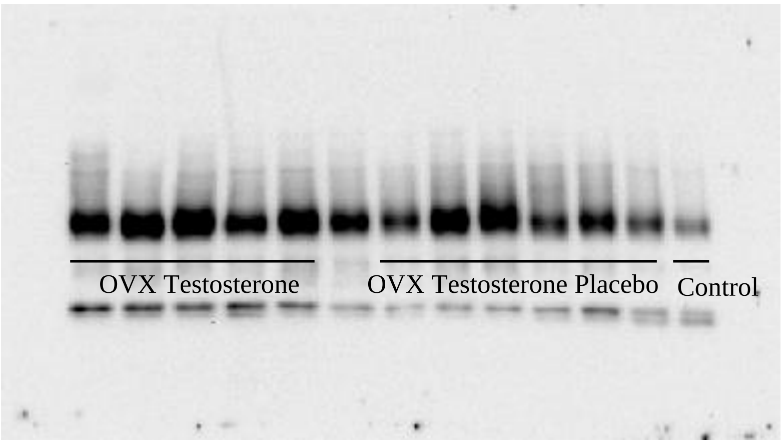

CST Testosterone, CST Testosterone Placebo

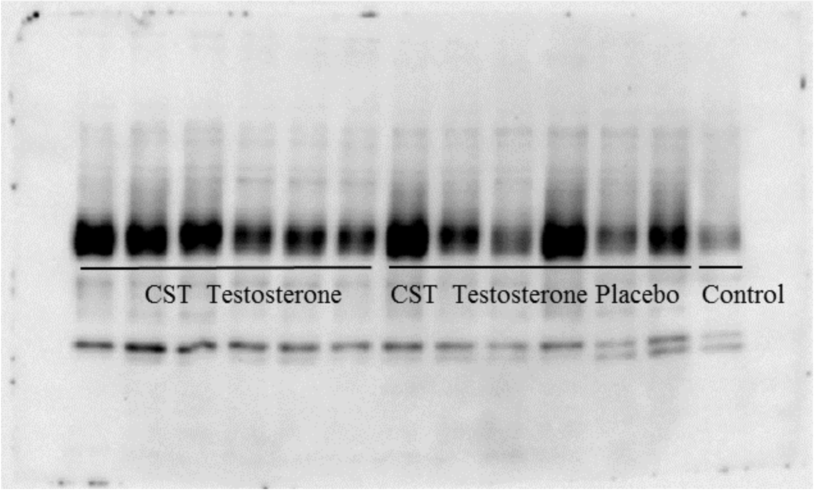

Male, Female

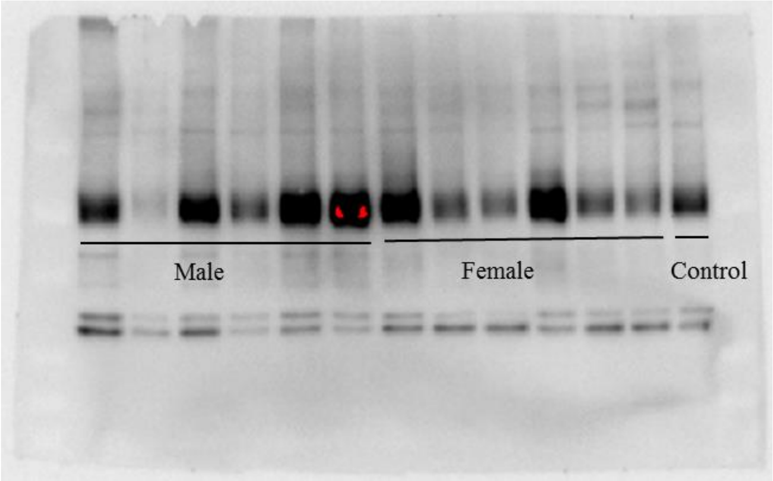

SMCT1

OVX Estrogen, OVX Estrogen Placebo

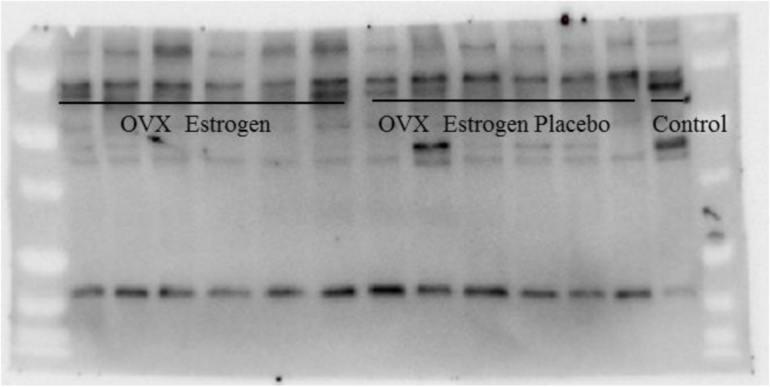

CST Estrogen, CST Estrogen Placebo

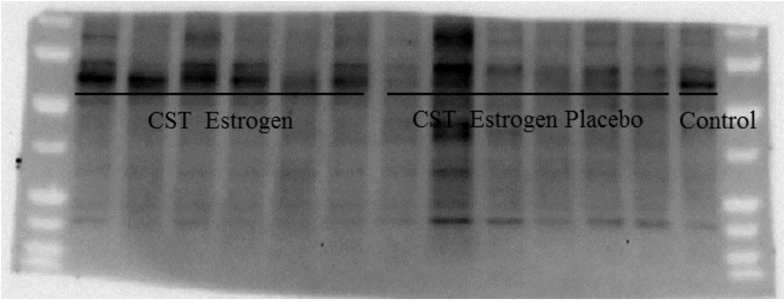

OVX Progesterone, OVX Progesterone Placebo

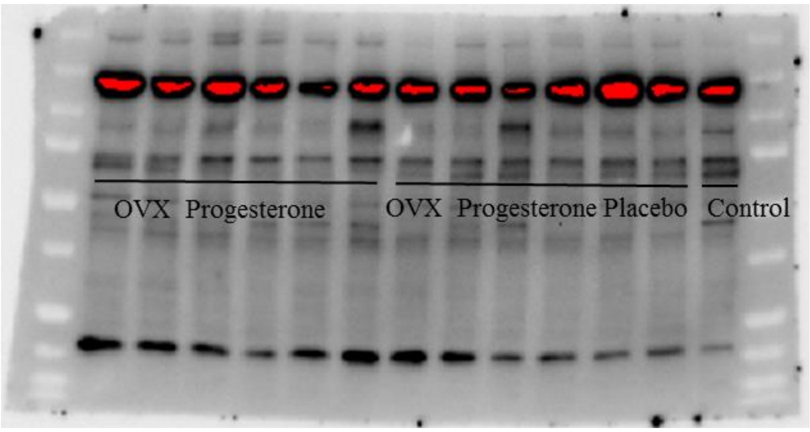

CST Progesterone, CST Progesterone Placebo

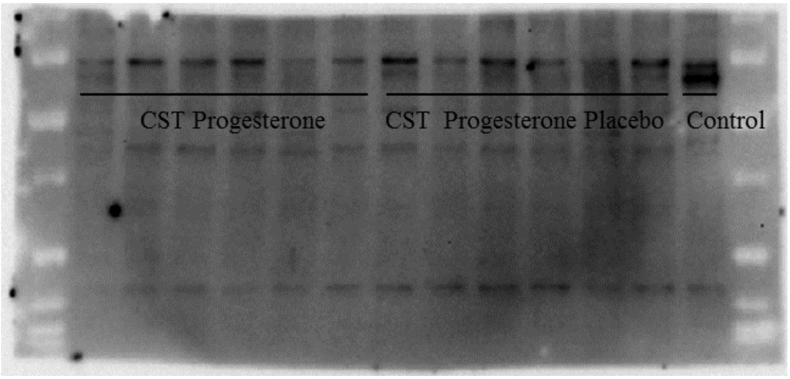

OVX Combo, OVX Combo Placebo

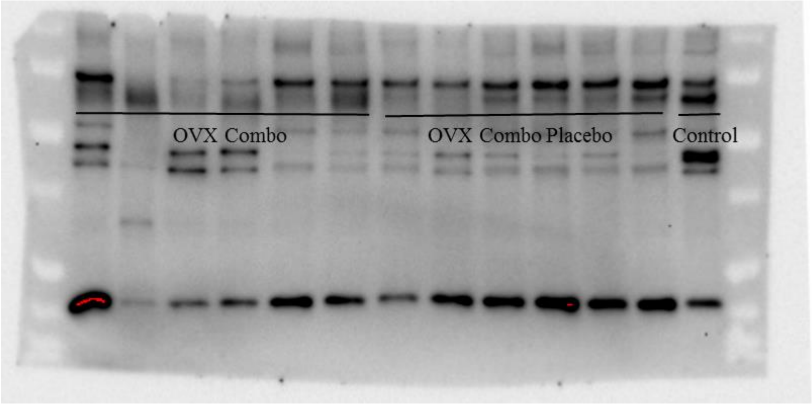

CST Combo, CST Combo Placebo

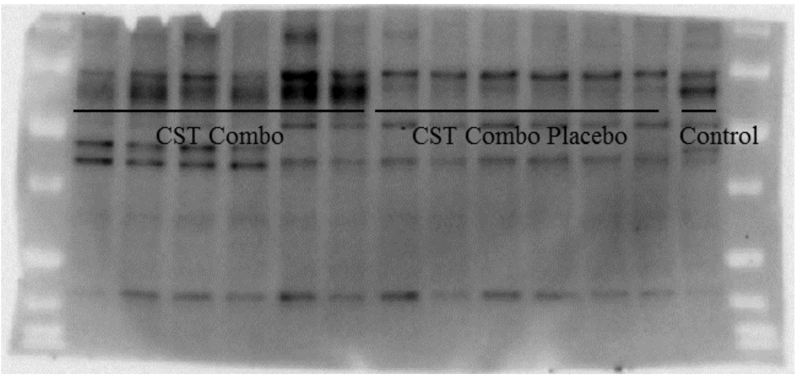

OVX Testosterone, OVX Testosterone Placebo

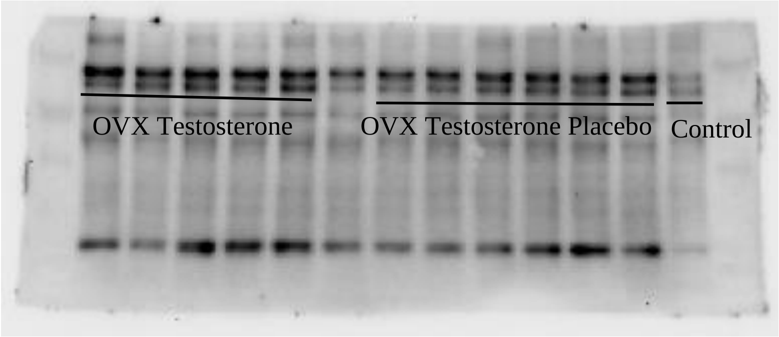

CST Testosterone, CST Testosterone Placebo

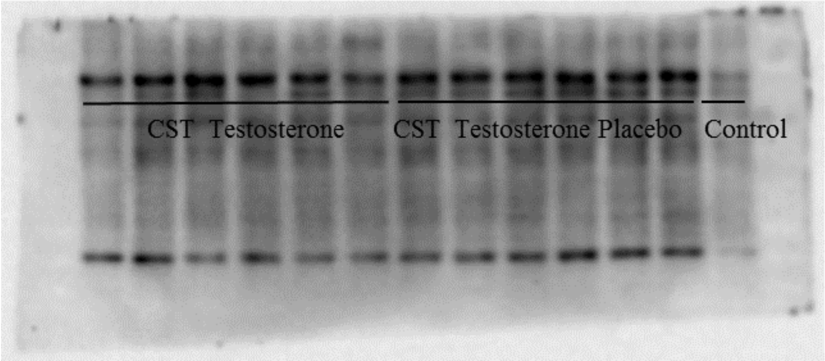

Male, Female

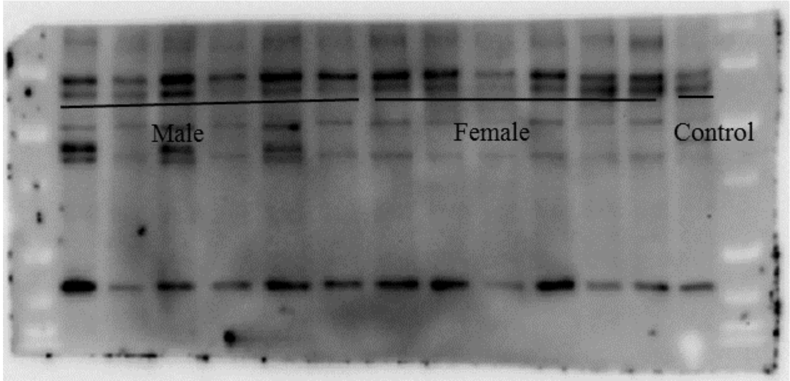

Supplement: Supplementary file 1 [file pharmaceutics-15-02404-s001.zip › pharmaceutics-2609983-Figure S1a.pdf]
