# Supplementary figures and images for: Effect of Sex and Cross-Sex Hormone Treatment on Renal Monocarboxylate-Transporter Expression in Rats
Source: Pharmaceutics. 2023 Sep 29;15(10):2404. doi: 10.3390/pharmaceutics15102404 (PMC10610497; doi:10.3390/pharmaceutics15102404)

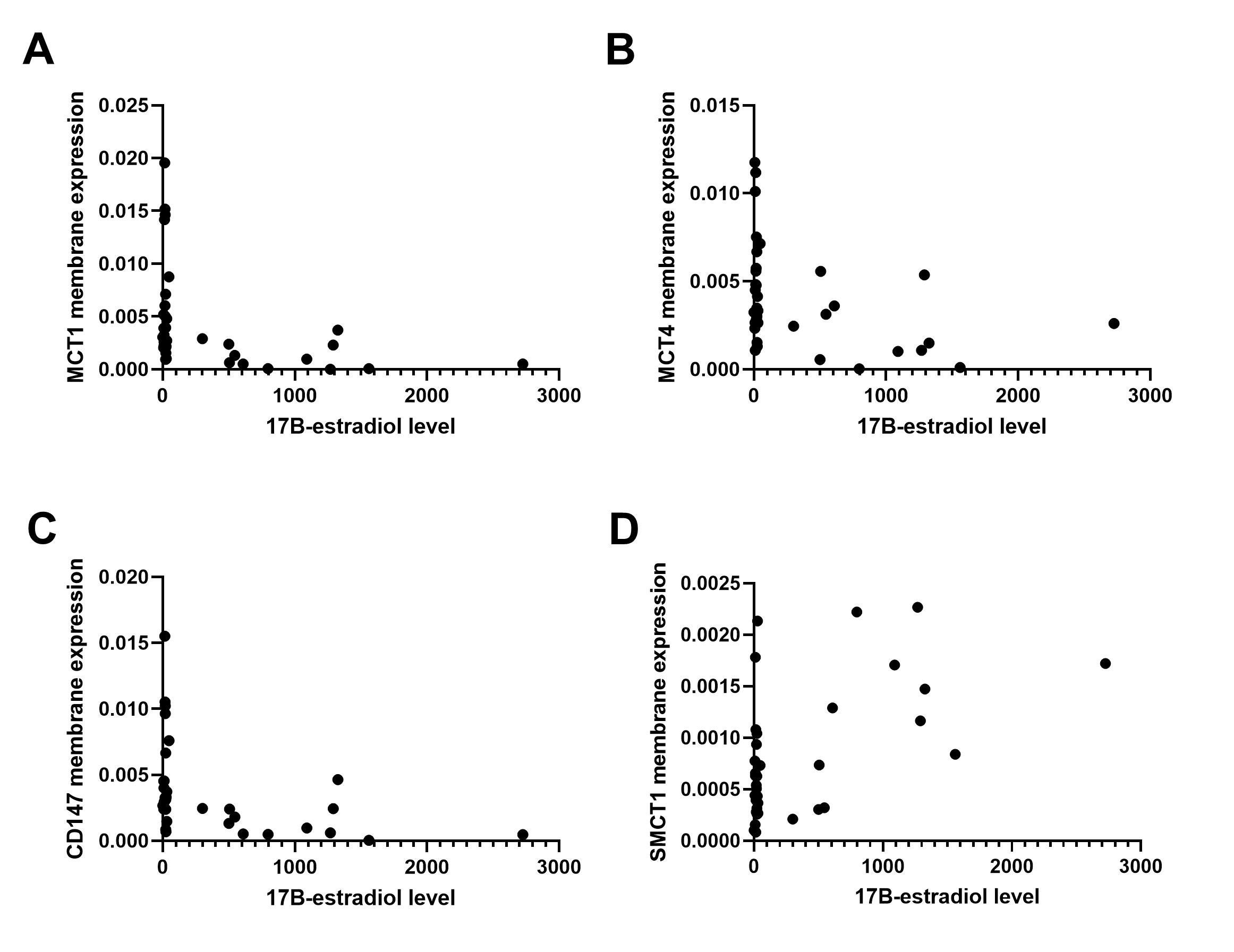

Supplement: Supplementary file 1 [file pharmaceutics-15-02404-s001.zip › Figure S2 Correlation - Estradiol.jpg]

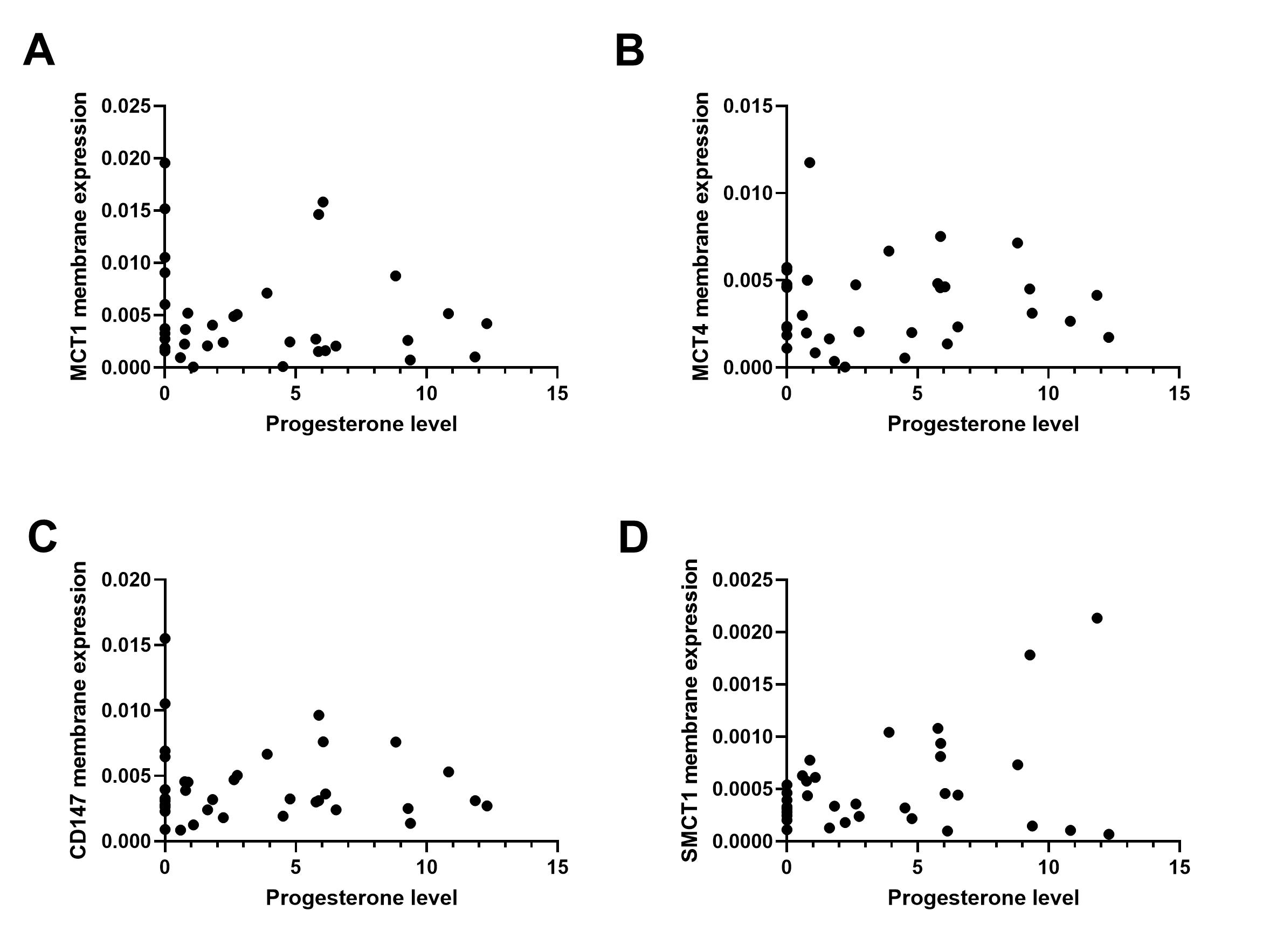

Supplement: Supplementary file 1 [file pharmaceutics-15-02404-s001.zip › Figure S3 Correlation - progesterone.jpg]

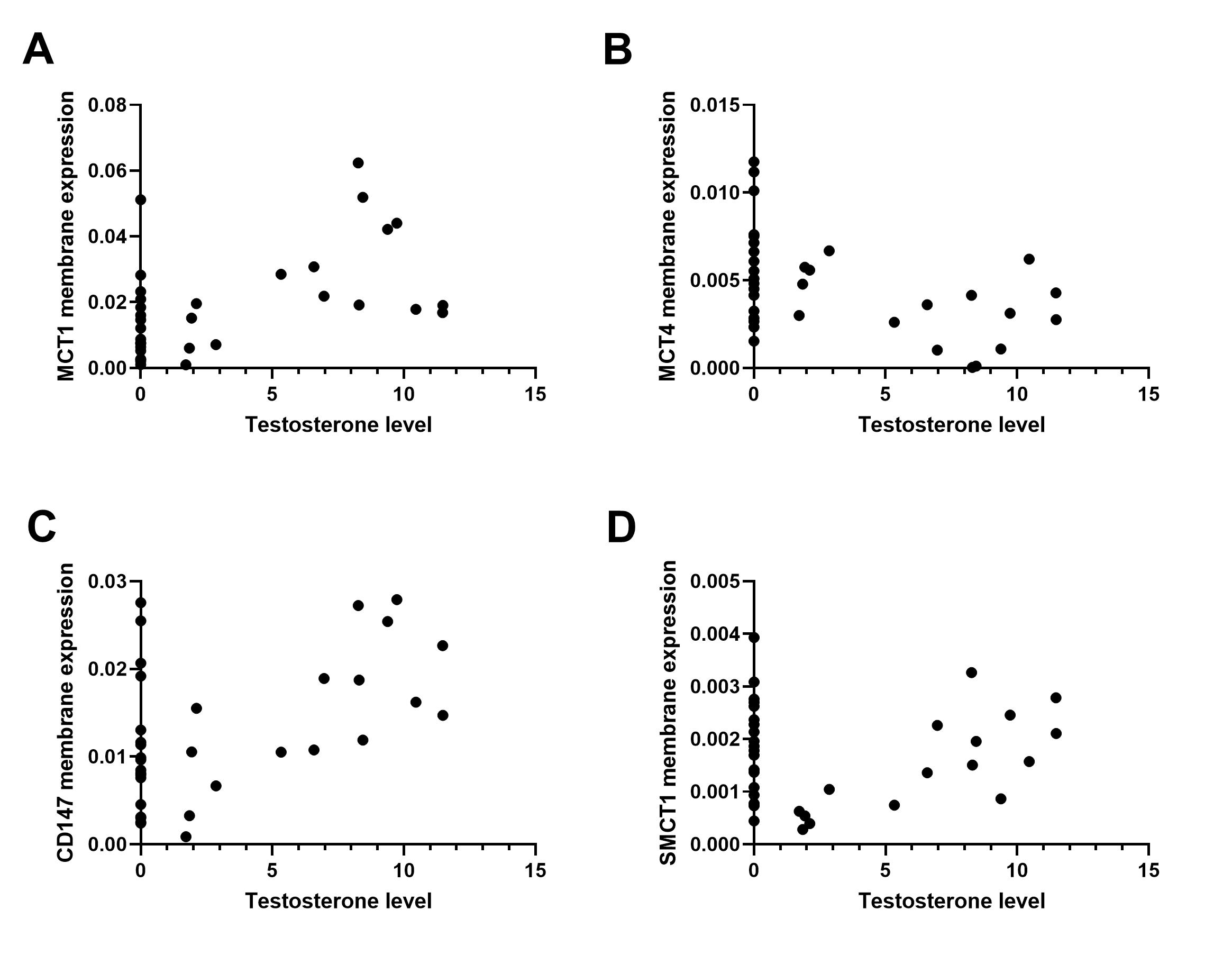

Supplement: Supplementary file 1 [file pharmaceutics-15-02404-s001.zip › Figure S4 Correlation - testosterone.jpg]
